# Effects of the VIVIFRAIL Exercise Protocol on Circulatory and Intracellular Peripheral Mediators Bridging Mitochondrial Dynamics and Inflammation in Robust and Frail Older People

**DOI:** 10.1111/acel.70029

**Published:** 2025-03-04

**Authors:** Fiona Limanaqi, Evelyn Ferri, Pasquale Ogno, Franca Rosa Guerini, Gabriela Alexandra Mihali, Tiziano Lucchi, Mario Clerici, Chiara Fenoglio, Laura D'Andrea, Elena Marcello, Mara Biasin, Beatrice Arosio

**Affiliations:** ^1^ Department of Biomedical and Clinical Sciences University of Milan Milan Italy; ^2^ IRCCS Fondazione Ca' Granda Ospedale Maggiore Policlinico Milan Italy; ^3^ IRCCS Fondazione Don Carlo Gnocchi ONLUS Milan Italy; ^4^ Asst Fatebenefratelli Sacco Milan Italia Italy; ^5^ Department of Pathophysiology and Transplantation University of Milan Milan Italy; ^6^ Department of Biomedical, Surgical and Dental Sciences University of Milan Milan Italy; ^7^ Department of Pharmacological and Biomolecular Sciences “Rodolfo Paoletti” University of Milan Milan Italy; ^8^ Department of Clinical Sciences and Community Health University of Milan Milan Italy

**Keywords:** apoptosis, ccf‐mtDNA, cytokines, exercise, inflammaging, mitochondria, mitophagy, peripheral blood cells

## Abstract

Physical exercise has been associated with healthier aging trajectories, potentially preventing or mitigating age‐related declines. This occurs through a complex, yet poorly characterized network of multi‐organ interactions involving mitochondrial, inflammatory, and cell death/survival pathways. Here, we comprehensively evaluated the 12‐week VIVIFRAIL multicomponent exercise protocol in physically frail (*n* = 16, mean age 81.4 ± 5.6) and robust (*n* = 50, mean‐age 73.6 ± 4.7) old individuals. Before (T0) and after (T1) the protocol, functional outcomes were assessed alongside a detailed exploratory analysis of mitochondrial, inflammatory, apoptotic, and neuro‐muscular mediators concerning their plasmatic/serum concentrations, and/or mRNA expression from peripheral blood mononuclear cells (PBMCs). Besides significant functional improvements across both groups, our findings highlighted unique and overlapping modulations of key biological pathways. Both groups showed refined mitochondrial integrity/turnover (upregulated *mt‐ND1*, downregulated *TFAM*, and *ULK1*), anti‐inflammatory responses (upregulated *IL10*, and *TGF‐B*, and downregulated *IL6/IL10* mRNA ratio), as well as reduced cellular damage/apoptosis (reduced plasmatic ccf‐nDNA, downregulated *BAX*, and upregulated *BCL‐2/BAX* ratio). Plasmatic ccf‐mtDNA was significantly reduced in robust subjects, while plasmatic IL6 and IL6/IL10 ratio were reduced in frail subjects uniquely. Spearman correlations between physical improvements and biological pathway variations also suggested different adaptation mechanisms influenced not only by chronological age but also by frailty status. In conclusion, this study confirms the benefits of physical activity in the older population and provides novel insights into specific biological mediators of the mitochondria‐inflammation axis as key players in such effects. Moreover, our findings establish PBMCs as a valuable tool for monitoring the biological trajectories of aging and health‐promoting lifestyle interventions.

## Graphical abstract

Physical activity promotes functional improvements in both robust and physically frail older adults, driven by distinct adaptation mechanisms in the mitochondria‐inflammation axis that are influenced by age and frailty status.

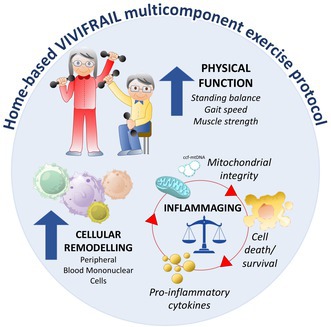

## 1 Introduction

Aging is an irreversible biological process characterized by a progressive loss of cellular functions and systemic deterioration of multiple tissues. This is accompanied by an overall decline in structural and functional body parameters, including physical and cognitive abilities, along with increased vulnerability to chronic conditions (López‐Otín et al. 2013). The clinical complexity featuring older people is best captured by the concept of frailty, a condition characterized by increased vulnerability to stressors and decreased homeostatic reserves, which in turn triggers a cascade of dysregulations culminating in reduced physical function and, ultimately, disability (Howlett, Rutenberg, and Rockwood 2021). Indeed, physical frailty is a predisability condition that, in the absence of targeted preventive interventions, may progress to sarcopenia (i.e., the age‐related skeletal muscle decline) (Cesari et al. 2014). Therefore, identifying and contrasting this condition at an early stage is a key to hindering the development of physical frailty and preventing its negative consequences (Morley et al. 2013). The implementation of physical performance assessment tools, in addition to those for measuring muscle mass, to objectively assess the vulnerability of older people to frailty is of paramount importance (Abizanda et al. 2012).

The biological underpinnings of physical frailty include exacerbated inflammatory response, mitochondrial alterations, and oxidative stress, up to immune dysfunction, which are a matter of intense investigation in a variety of body fluids, tissues, and cells (Finkel and Holbrook 2000; Soto et al. 2023; Soysal et al. 2016).

Among the mechanisms potentially having a role in the pathophysiology of frailty, there is inflammaging, the chronic, systemic, low‐grade pro‐inflammatory state characterizing the aging process (Franceschi et al. 2006). Inflammaging can be seen not only as a harmful phenomenon but also as a positive adaptive mechanism in response to the stimuli to which the individual is subjected throughout life. An association between frailty and increased concentrations in peripheral blood of C‐reactive protein (CRP), interleukin (IL)‐6, tumor necrosis factor‐α (TNF‐ α), and its receptor TNFR1 was described in older subjects (Cesari et al. 2004; Soysal et al. 2016; Xu et al. 2022).

An intimate crosstalk exists between inflammation and mitochondrial dynamics, which contributes to the pathophysiology of aging (López‐Armada et al. 2013). Beyond muscle and nerve cells, this is relevant for immune cells as well, which require high energy demand that is regulated by mitochondria. Again, immune cells are highly metabolically flexible, reprogramming their mitochondrial function upon inflammatory activation to meet their energy demands (Ehinger et al. 2024). Pro‐inflammatory mediators may promote a vicious cycle of cell danger/death pathways (e.g., inflammasomes, caspases, and redox stress) that contribute to altering nuclear and mitochondrial DNA integrity and copy number (Yu et al. 2014). In turn, compromised mitochondrial dynamics orchestrate the production of reactive oxygen species, as well as constitutive and metabolic products acting as damage‐associated molecular patterns (DAMPs) that, when released into the cytosol or extracellular milieu, exacerbate inflammation and cell dysfunction (Little et al. 2014; Mittal et al. 2014; Shimada et al. 2012). This is the case of mitochondrial DNA fragments (circulating cell‐free mitochondrial DNA, ccf‐mtDNA), mtDNA‐binding proteins (e.g., TFAM), and also nuclear DNA fragments (circulating cell‐free nuclear DNA, ccf‐nDNA), which are widely explored as markers of cellular stress and inflammation in many age‐related conditions (Gambardella et al. 2019; Nidadavolu et al. 2023; Picca et al. 2019).

Persistent inflammatory states may contribute to both physical and cognitive decline by affecting muscle homeostasis while concurrently promoting neuroinflammation and/or neuronal dysfunction (Lavine and Sierra 2017). In chronic conditions, including frailty, the modulation of the muscle‐brain axis is best represented by the release of myokines, neurotrophins, and/or neuromuscular junction mediators that are known to regulate neuronal as well as muscle homeostasis, such as Irisin (Korta et al. 2019), Brain‐Derived Neurotrophic factor (BDNF) (Roh et al. 2022), C‐Terminal Agrin Fragment (CAF) (de Souza Ramos et al. 2022), Neurofilament Light chain (NfL) (Khalil et al. 2020), and Cyclase‐Associated Protein 2 (CAP2) (Pelucchi et al. 2023). These pieces of evidence highlight the need for integrated interventions targeting both physical and cognitive health to effectively address and mitigate frailty. In this frame, appropriate and regular exercise or increased mobility is shown to improve body organic conditions and to maintain functional capacity while slowing down age‐related physical decline (Distefano et al. 2018; Petrella et al. 2021). This occurs through a complex, yet not fully characterized network of interactions, which involves changes in immune, mitochondrial, and muscle‐brain mediators, among others.

The VIVIFRAIL multicomponent physical protocol is an exercise intervention program consisting of resistance training, gait re‐training, and balance training, and appears to be the best strategy for improving gait, balance, and strength, as well as for reducing the rate of falls (Izquierdo 2019). This type of intervention is performed according to the functional capacity and has proven safe and effective in reversing the functional decline associated with acute hospitalization in very old people (Martínez‐Velilla et al. 2019).

In the present study, we aimed to investigate in depth whether, and how the VIVIFRAIL protocol modifies systemic inflammatory, mitochondrial, and apoptotic pathways, along with mediators of the muscle–brain axis in robust and physically frail older individuals.

## 2 Materials and Methods

### 2.1 Enrollment, Physical Intervention, and Functional Assessment

Sixty‐six individuals (24 males and 42 females) were enrolled by the Fondazione IRCCS Ca’ Granda, Ospedale Maggiore Policlinico, Milan, Italy. Physical function was determined by means of the Short Physical Performance Battery (SPPB), which assesses standing balance, gait speed, and muscle strength (0, worst performance to 12 points, best performance). The SPPB of the individuals with physical frailty ranged between 3 and 9 and was > 9 in robust subjects (Marzetti et al. 2018). All subjects were also characterized for the Tinetti score (Tinetti 2003) and the left and right Handgrip (HG) test (Sousa‐Santos and Amaral 2017) to assess functional status, and for a modified version of the Mini‐Mental State Examination (MMSE) (Magni et al. 1996) to assess cognitive status. According to the SPPB scores and the fall risk, subjects underwent a home‐based, low‐cost, and customized multicomponent physical exercise program (Program D, exercises for subjects with no limitations; Program C and B, exercises for subjects with slight or moderate limitations), as previously described (Agostini et al. 2023; Petrella et al. 2021). In brief, the VIVIFRAIL multicomponent exercise protocol (Izquierdo 2019), including arm and leg strength and power exercises, balance and coordination to prevent falls, flexibility, and cardiovascular endurance exercises, lasted 12 weeks. The SPPB, Tinetti, HG, and MMSE were assessed after the VIVIFRAIL protocol as well. Inclusion criteria were age > 60 years, sufficient cognitive abilities to provide informed consent to the study and to participate and follow the protocol (MMSE ≥ 24), and ability to walk in autonomy. Exclusion criteria were localized loss of strength and aphasia due to severe stroke, severe impairment of motor skills, steroid therapy, and concomitant diagnosis of neoplastic or neurodegenerative diseases.

### 2.2 Quantification of Inflammatory and Muscle–Brain axis' Mediators in Plasma

Peripheral blood was collected from each subject before (T0) and after the VIVIFRAIL protocol (T1) in Vacuette tubes (Greiner Bio‐One, Kremsmünster, Austria). Platelet‐free plasma and serum were obtained from blood centrifugation at 1200 *g* for 15 min at room temperature (RT) and stored at −80°C until testing. The plasmatic concentrations of IL‐6, IL‐10, IL‐1b, TNF‐a, Interferon gamma (IFN‐g), TNFR1, soluble Triggering receptor expressed on myeloid cells 1 and 2 (sTREM1, sTREM2), BDNF, and NfL were assessed by using the Human Simple Plex assays (ProteinSimple, Bio‐Techne, CA, USA) on the Ella instrument (ProteinSimple, Bio‐Techne, CA, USA), according to the manufacturer's instructions. Plasma concentrations of Irisin were measured with the Human Irisin/FNDC5 enzyme‐linked immunosorbent assay (ELISA) kit (NOVUS Biologicals, Bio‐Techne, Minneapolis, Minnesota, USA). Serum concentrations of CAF were assessed with the Human CAF ELISA kit (BT Laboratory, Shanghai, China). Serum CAP2 concentration was determined using a commercially available ELISA kit (n. IK5163; Immunological Sciences) (Pelucchi et al. 2023).

### 2.3 Isolation and Quantification of ccf‐mtDNA and ccf‐nDNA in Plasma

DNA from platelet‐free plasma was isolated through the ReliaPrep Viral TNA Miniprep Custom kit via a column‐based method (PROMEGA, Madison, Wisconsin, USA). Briefly, 250 μL of plasma were mixed with 25 μL of protein kinase, and 250 μL of cell lysis buffer (PK, A505F, and CLD A506C, PROMEGA, Madison, Wisconsin, United States), and incubated at 56°C for 30 min to ensure the lysis of extracellular vesicles potentially containing nucleic acids. Then, 250 μL of binding buffer (BBA, A502C, PROMEGA, Madison, Wisconsin, United States) was added, and the mixture was transferred to the Binding Column. Samples were centrifuged at maximum speed for 1 min at RT. The DNA retained by the column was washed thrice with 500 μL of Column Wash Solution (CWD A503C, PROMEGA, Madison, Wisconsin, USA) followed by centrifugation at maximum speed for 3 min at RT. Finally, the column was transferred to a clean 1.5 mL microcentrifuge tube, and DNA was eluted in 60 μL of Elution Buffer through centrifugation at maximum speed for 1 min at RT. mtDNA and nDNA copy numbers were measured via quantitative real‐time PCR (qPCR, CFX96 connect, Bio‐Rad, Hercules, CA, USA) through the SsoAdvanced Universal SYBR Green Supermix (Bio‐Rad, Hercules, CA, USA), according to the following thermal profile: 95°C for 10 min (initial denaturation), followed by 45 cycles at 94°C for 30 s (denaturation), 60°C for 60 s (annealing), and 72°C for 7 s (extension). Already optimized primers (PrimePCR SYBR Green Assay Bio‐Rad, Hercules, CA, USA) specific for the nDNA (ribosomal protein, large, P0, RPLP0) and for mtDNA (mitochondrially encoded NADH dehydrogenase 1, mt‐ND1) were used for quantification by referring to standard curves generated by using 20 million copies of template diluted 10‐fold to 20 copies for each target (PrimePCR Template for SYBR Green Assay, Bio‐Rad, Hercules, CA, USA). qPCR was performed in a total reaction volume of 10 μL, containing 1 μL of primers, 5 μL of SYBR Green Supermix, and 4 μL of DNA. The ccf‐nDNA and ccf‐mtDNA copy numbers were expressed as copies/μL of plasma. Each qPCR was performed in triplicate, along with negative controls (Elution Buffer) and positive controls (cDNA from PBMCs). All the reactions were followed by melting curve analysis. Results are shown as gene copies/μL of plasma.

### 2.4 RNA Extraction From PBMCs, cDNA Reverse Transcription, and Quantification Through Real‐Time qPCR

PBMCs were isolated through density gradient centrifugation on Ficoll (Lympholyte‐H, Cedarlane Laboratories Limited, Hornby, ON, Canada) and stored at −80°C until use. RNA extraction from PBMCs was performed using Chomczynski and Sacchi's modified method (Chomczynski and Sacchi 2006). RNA was dissolved in RNase‐free water and quantified by the NanoPhotometer N60 (Implen GmbH, München, Germany). Two μg of total RNA was reverse transcribed using the SuperScript VILO cDNA Synthesis Kit (Invitrogen by Thermo Fisher Scientific, Massachusetts, United States). Twenty‐five ng of cDNA were amplified with either designed or already customized primer pairs (PrimePCR SYBR Green Assay, Bio‐Rad) by real‐time qPCR (CFX96 connect, Bio‐Rad, Hercules, CA, USA) through Universal SYBR Green Supermix (Bio‐Rad, Hercules, CA, USA), according to the following thermal profile: 95°C at 15 min (initial denaturation), 15 s at 95°C (denaturation), 1 min at 60°C (annealing) and 20 s at 72°C (extension) for 40 cycles. Negative controls were included in each run. Each PCR was performed in triplicate. Melting curves were analyzed for amplicon characterization. Results for gene expression were calculated by the 2^−ΔΔCt^ equation. Results are normalized to the *GAPDH* housekeeping gene and shown as relative mRNA expression units (in percentage) to an internal reference sample (a.u. 100%). The analyzed genes partaking in (i) mitochondrial dynamics/mitophagy, intrinsic apoptosis, and oxidative metabolism and (ii) inflammation and extrinsic apoptosis are listed in Table S1. Sequences of the designed primers are listed below: **GAPDH:** Forward 5′‐3′ CGGATTTGGTCGTATTGGG; Reverse 5′‐3′ GCTTCCCGTTCTCAGCCTTG; **IL‐10:** Forward 5′‐3′ CTCCACGGCCTTGCTCTTGT; Reverse 5′‐3′ TCAAGGCGCATGTGAACTCC; **IL‐6:** Forward 5′‐3′ GGTGTTGCCTGCTGCCTTC; Reverse 5′‐3′ GCCAGTGCCTCTTTGCTGCT. The remaining primers were purchased as already customized (PrimePCR SYBR Green Assay, Bio‐Rad).

### 2.5 Statistical Analyses

Normal distribution was tested through the Kolmogorov–Smirnov test. Normally distributed values were reported as mean ± standard deviation (SD), while nonnormally distributed data were reported as median and interquartile range (IQR: 25‐75th percentile). Categorical data were compared using Pearson's chi‐square test. The normally distributed variables were analyzed using the *Student's t*‐test. Not‐normally distributed data were analyzed using the Wilcoxon matched‐pairs signed rank (for within‐group repeated measures, T0 vs. T1) and the Mann Whitney test (for nonrepeated comparisons, robust vs. frail, males vs. females). Spearman's correlation was assessed to investigate associations between clinical and functional variables, gene expression, and blood concentrations of each component. The *p* values corresponding to ≤ 0.05 were considered statistically significant. Statistical analyses were performed using the commercial software GraphPad Prism (Version 5, La Jolla, CA, USA) and IBM SPSS Statistic (version 28, IBM Inc., Chicago, IL, USA).

## 3 Results

### 3.1 Demographic and Clinical Characterization of the Study Cohort Before and After the VIVIFRAIL Exercise Protocol

Demographic and clinical characteristics of the study population before (T0) and after (T1) the VIVIFRAIL exercise protocol are shown in Table 1. Sex was similarly distributed in the two groups, while the frail subjects were older than the robust ones (*p* < 0.001). As per definition, SPPB and Tinetti scores were significantly lower in frail compared to robust subjects before (T0) the VIVIFRAIL protocol (*p* < 0.001 for both comparisons). At T0, left and right HG scores were the only parameters that significantly differed in males compared to females. When considering robust and frail subjects independently of sex, at T0, left and right HG scores did not significantly differ between the two groups (Table 1). However, when stratifying according to sex, left and right HG scores at T0 were significantly lower in frail compared to robust females, while only right HG was lower in frail compared to robust males. MMSE scores were in the normal range and did not differ between robust and frail subjects. As expected, physical intervention produced significant beneficial effects in both frail and robust older people. Following the VIVIFRAIL protocol, SPPB score was significantly improved in both robust and frail groups (*p* < 0.001). Starting from a lower SPPB value (Table 1), the frail subjects showed a more pronounced increase in this parameter, as supported by the significantly higher variation of SPPB compared to the robust subjects independently of sex (ΔSPPB: T1‐T0, *p* < 0.001) (Figure S1A,B). The SPPB remained significantly lower in frail compared to robust subjects even after (T1) the exercise protocol (*p* < 0.001) (Table 1). Tinetti score was also significantly improved after physical exercise, but only in frail subjects (*p* < 0.01). The left and right HG scores were increased both in the robust (*p* < 0.0001) and frail subjects (*p* > 0.01), in both males and females (*p* < 0.001 and *p* < 0.01 robust males, *p* < 0.0001 robust females, *p* < 0.05 frail males, *p* < 0.001 frail females). Variations in left HG scores were higher in males compared to females (*p* < 0.05 robust, *p* = 0.056 frail) (Figure S1C). At T1, left and right HG scores remained significantly lower in frail compared to robust females (*p* < 0.05 for left HG, *p* < 0.01 for right HG). Though in the normal range, the MMSE slightly, and similarly increased in both groups, but it reached statistical significance in the robust subjects only (*p* < 0.05 vs. T0) (Table 1).

**TABLE 1:** Demographic and clinical characteristics of robust and frail subjects before (T0) and after (T1) the VIVIFRAIL© protocol. Age is shown as mean ± SD. SPPB, Tinetti, left and right HG scores, and MMSE are shown as median (IQR: 25‐75th percentile).

	Robust subjects					Frail subjects				
*n*	50					16				
Sex (F:M)	33:17					9:7				
Age	73.6 ± 4.7					81.4 ± 5.6 ^### vs Robust^				
	Pre‐ VIVIFRAIL© (T0)		Post‐VIVIFRAIL© (T1)		*p* T0 vs T1	Pre‐VIVIFRAIL© (T0)		Post‐VIVIFRAIL© (T1)		*p* T0 vs T1
SPPB	11.0 (11.0–12.0)		12.0 (12.0–12.0)		***	8.0 (7.2–9.0) ### vs. Robust		10.0 (9.0–11.0) ^### vs Robust^		***
Tinetti	28.0 (27.0–28.0)		28.0 (27.0–28.0)		ns	26.0 (25.0–27.0) ^### vs Robust^		28.0 (27.0–28.0) ^ns vs Robust^		**
Left HG	19.6 (16.7–26.0)		25.0 (20.9–33.2)		****	17.5 (15.0–19.5) ^ns vs Robust^		18.7 (16.2–26.9) ^ns vs Robust^		**
	M 27.0 (25.0–31.0)	F 18.0 (16.0–20.75)	M 34.0 (30.0–40.5)	F 20.0 (17.0–24.0)	M*** F****	M 20.0 (18.0–30.0) ns vs. Robust M	F 15.0 (12.50–16.50) ## vs. Robust F	M 32 (24.0–40.0) ns vs. Robust M	F 16.75 (14.42–19.33) # vs. Robust F	M* F***
Right HG	22.5 (18.0–29.2)		25.7 (18.7–32.0)		****	17.0 (16.0–24.7) ^ns vs Robust^		18.2 (16.0–25.5) ^ns vs Robust^		**
	M 31.0 (28.50–33.0)	F 19.0 (16.0–23.0)	M 34.0 (30.0–40.50)	F 22.0 (18.0–25.75)	M** F****	M 20.0 (18.0–30.0) ^# vs Robust^	F 15.0 (12.5–16.5) ## vs. Robust F	M 32.0 (24.0–40.0) ns vs. Robust M	F 16.75 (14.42–19.33) ## vs. Robust F	M* F***
MMSE	29 (28.75–30.0)		29 (29.0–30.0)		*	28.5 (27.25–30.0)		28.5 (28.0–30.0) ^ns vs Robust^		ns

*Note:* #*p* < 0.05, ##*p* < 0.01, and ###*p* < 0.001 frail versus robust subjects; *p < 0.05, **p < 0.01, ***p < 0.001, and ****p < 0.0001 T0 versus T1; ns: Nonsignificant.

Abbreviations: F, females; HG, Handgrip; M, males; MMSE, Mini‐Mental State Examination; SPPB, Short Physical Performance Battery.

### 3.2 Correlations Among Clinical Scores and Biological Mediators Before the Physical Exercise Protocol (T0)

**FIGURE 1:**
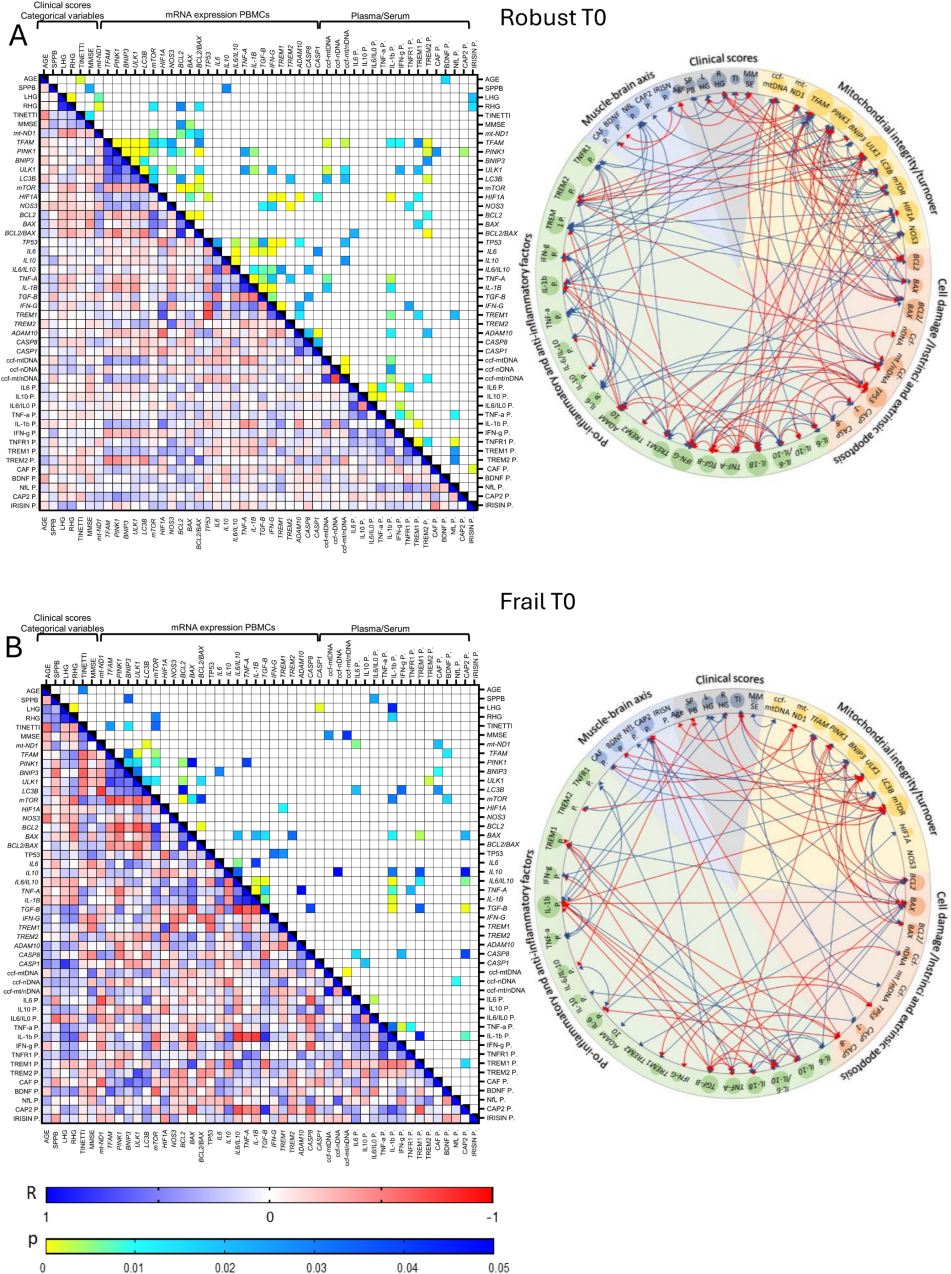
Spearman's correlations among categorical variables, clinical scores, and mitochondrial, apoptotic, inflammatory, and muscle–brain axis' mediators in robust (A) and frail (B) individuals before the physical exercise protocol (T0). Heatmaps show Spearman's R correlations and related *p* values. The cartoons provide a summary of positive (blue), and negative (red) inter‐ and intra‐pathway associations. LHG, Left Handgrip; RHG, Right Handgrip; TI, Tinetti.

At baseline (T0), several associations emerged among age, physical scores, and the analyzed biological mediators (Figure 1). In robust subjects (Figure 1A), age was negatively associated with (i) Tinetti score (*R* = −0.43, *p* < 0.01) and (ii) plasmatic BDNF (*R* = −0.32, *p* < 0.05). SPPB was positively correlated with PBMCs' mRNA of *IL‐10* (*R* = 0.28, *p* < 0.05). Left and/or Right HG scores were (i) negatively correlated with PBMCs' mRNA of *mt‐ND1* (*R* = −0.38 and−0.45, *p* < 0.01), *mTOR* (*R* = −0.34, *p* < 0.05), *Bcl‐2* (*R* = −0.28, *p* < 0.05), and *Bcl‐2/BAX* mRNA ratio (*R* = ‐0.33, *p* < 0.05) and (ii) positively associated with the plasmatic Irisin (*R* = 0.31, *p* < 0.05). Finally, MMSE was associated with (i) SPPB (*R* = 0.30, *p* < 0.05) and Tinetti (*R* = 0.35, *p* < 0.05) and (ii) mRNA expression of *Bcl‐2* (*R* = −0.29, *p* < 0.05) and *BAX* (*R* = −0.30, *p* < 0.01). As for inter‐ and intra‐pathway associations, mitochondrial integrity/turnover and oxidative stress genes measured from PBMCs were the most represented ones (*TFAM*, *PINK1*, *ULK1, HIF1A*), followed by PBMCs' mRNA expression and/or plasmatic concentrations of cell damage/apoptosis (*Bcl‐2/BAX* ratio, *TP53*, ccf‐mt/nDNA) and inflammatory mediators (*IL‐1b*, *IFN‐g*, sTREM2, sTREM1, and sTNFR1). In robust subjects specifically, positive correlations between mRNA of *TFAM*, *ULK1*, and *LC3B* with plasmatic ccf‐mtDNA copies were observed as well (Figure 1A and File S1). Interactions involving mediators of the muscle‐brain axis were overall poorly represented, despite moderate associations of plasmatic NfL with some inflammatory mediators (*TREM1*, TNF‐a, sTNFR1, sTREM1, sTREM2).

In frail individuals (Figure 1B), age was negatively associated with the Tinetti score (*R* = −0.57, *p* < 0.05). SPPB was negatively associated with (i) PBMC's mRNA of *BNIP3* (*R* = −0.54, *p* < 0.05), and (ii) plasmatic IL‐6/IL‐10 ratio (*R* = −0.57, *p* < 0.05). Left and/or right HG strength scores were (i) negatively associated with the PBMCs' mRNA of *mTOR* (*R* = −0.55, *p* < 0.05), and (ii) positively associated with the PBMCs' mRNA of *CASP‐1* (*R* = 0.79 and 0.45, *p* < 0.001), and plasmatic IL‐1b (R = 0.59 and 0.51, *p* < 0.05). Tinetti was associated (i) negatively with the PBMCs' mRNA of *TFAM* (*R* = −0.58, *p* < 0.05) and *BNIP3* (*R* = ‐0.63, *p* < 0.05), and (ii) positively with the PBMCs' mRNA of *mTOR* (*R* = 0.56, *p* < 0.05). Finally, MMSE was associated positively with the plasmatic ccf‐mtDNA (*R* = 0.56, *p* < 0.05) and the ccf‐mt/nDNA ratio (*R* = 0.52, *p* < 0.05). A positive correlation of MMSE with Tinetti, which approached statistical significance (*R* = 0.50, *p* = 0.057), was detected as well.

Fewer but stronger, inter‐ and intra‐pathway interactions emerged in frail subjects compared to the robust ones (Figure 1B). Mitochondrial integrity factors remained the most represented (PBMCs' mRNA of *mTOR, BNIP3, PINK1, ULK1, LC3B*), followed by apoptotic (PBMCs' mRNA of *Bcl2*, and *BAX*), and inflammatory markers (plasmatic IL‐1b, and sTREM1, and PBMC's mRNA expression of *TGF‐b*, *TNF‐a*, *IL‐1b*, *IL‐6/IL‐10* ratio). Contrarily to robust subjects, several mediators of the muscle‐brain axis, including serum CAF, and CAP2, as well as plasmatic BDNF, were moderately represented in the frail group at baseline, showing associations with mediators of mitochondrial/apoptotic pathway (*mt‐ND1, TFAM, BNIP3, LC3B, mTOR, BAX*), and inflammatory pathway (*IL‐10*, *IL‐6/IL‐10*, *TGF‐B*, *CASP8*, IL‐1b, TREM1).

In both robust and frail subjects, positive correlations were generally observed among the mRNA expressions of mitochondrial pathway mediators, except for *mt‐ND1* and *mTOR* genes, which were negatively associated with *TFAM*, *ULK1*, and *LC3B* (Figure 1A,B and File S1). Again, in both groups, the mRNA expressions of most inflammatory mediators, including *IL‐6*, *IL‐10*, *IL‐6/IL‐10* ratio, *IL‐1b*, *IFN‐g*, and *TREM2*, showed no correlations with their plasmatic counterparts (Figure 1A,B). Instead, *IL‐1b* and *TREM1* mRNAs were negatively and positively associated with their plasmatic counterparts, respectively (robust IL‐1b: *R* = −0.35, *p* < 0.01, robust TREM1: *R* = 0.34, *p* < 0.05; frail IL‐1b: *R* = −0.77, *p* < 0.01).

### 3.3 Effects of VIVIFRAIL Exercise Protocol on Mitochondrial/Cell Stress Pathway and Intrinsic Apoptosis

**FIGURE 2:**
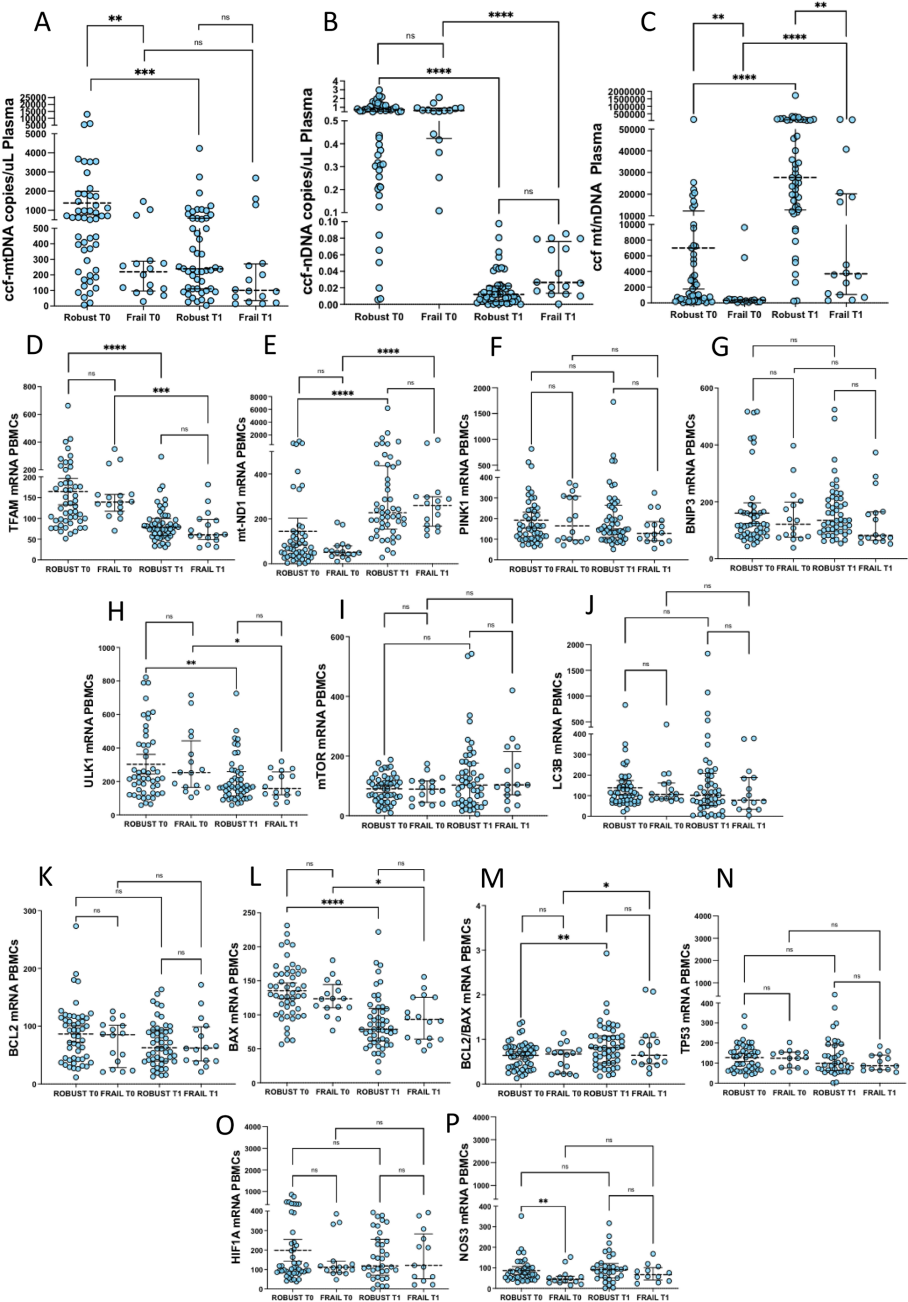
Effects of the VIVIFRAIL exercise protocol on plasmatic ccf‐DNA copies and relative PBMC's mRNA expression of biological mediators partaking in mitochondrial integrity/turnover, cell damage/danger, intrinsic apoptosis, and oxidative metabolism. Data were analyzed using the Wilcoxon matched‐pairs signed rank test (within‐group repeated measures, T0 vs. T1) and the Mann–Whitney test (Robust vs. Frail). Values are shown as median and interquartile range (IQR: 25‐75th percentile). *p < 0.05, **p < 0.01, ***p < 0.001, ****p < 0.0001.

Ccf‐mtDNA was significantly lower in frail compared to robust individuals before the physical intervention (*p* < 0.01, T0) (Figure 2A). Following the VIVIFRAIL protocol (T1), ccf‐mtDNA was significantly reduced in robust subjects only (*p* < 0.0001) (Figure 2A). In line with this, ccf‐mtDNA variation differed significantly between robust and frail subjects (Figure S2). Ccf‐nDNA was almost abolished in both robust and frail groups at T1 (*p* < 0.0001 for both robust and frail) (Figure 2B), and consequently, the ccf‐mt/nDNA ratio was potently increased (*p* < 0.0001 for both robust and frail) (Figure 2C). At the level of PBMC's mRNA, physical exercise produced a significant decrease of *TFAM* (robust *p* < 0.0001, and frail *p* < 0.001) (Figure 2D), paralleled by an increase of *mt‐ND1* (*p* < 0.0001 in both robust and frail) (Figure 2E). As for the expression of autophagy/mitophagy‐related genes (Figure 2F–J), significant changes were observed for *ULK1* only, which was decreased in both robust and frail individuals (robust *p* < 0.01, and frail *p* < 0.05) (Figure 2H). Despite an upward trend for *mTOR* observed in both groups (Figure 2I), no significant changes were observed for the expression of other autophagy/mitophagy‐related markers. Again, as for the mitochondrial‐related, anti‐ and pro‐apoptotic genes (Figure 2K–M), physical activity decreased the mRNA of *BAX* (robust *p* < 0.0001, and frail *p* < 0.05), meanwhile increasing that of the *Bcl‐2/BAX* ratio (robust *p* < 0.01, and frail *p* < 0.05) (Figure 2L,M). Interestingly, the *Bcl‐2/BAX* ratio, which was significantly associated with HG at T0, was higher in robust and frail females compared with robust and frail males, respectively (*p* < 0.05 for both comparisons) (Figure S3A). Again, after physical activity, the *Bcl‐2/BAX* ratio increased in robust females (*p* < 0.01) and in frail males (*p* < 0.01) (Figure S3A) suggesting possible sex‐related associations.

Finally, *NOS3* mRNA was lower in frail compared to robust individuals before (T0, *p* < 0.01) but not after (T1) physical exercise (Figure 2P).

### 3.4 Effects of VIVIFRAIL Exercise Protocol on Inflammatory Mediators

**FIGURE 3:**
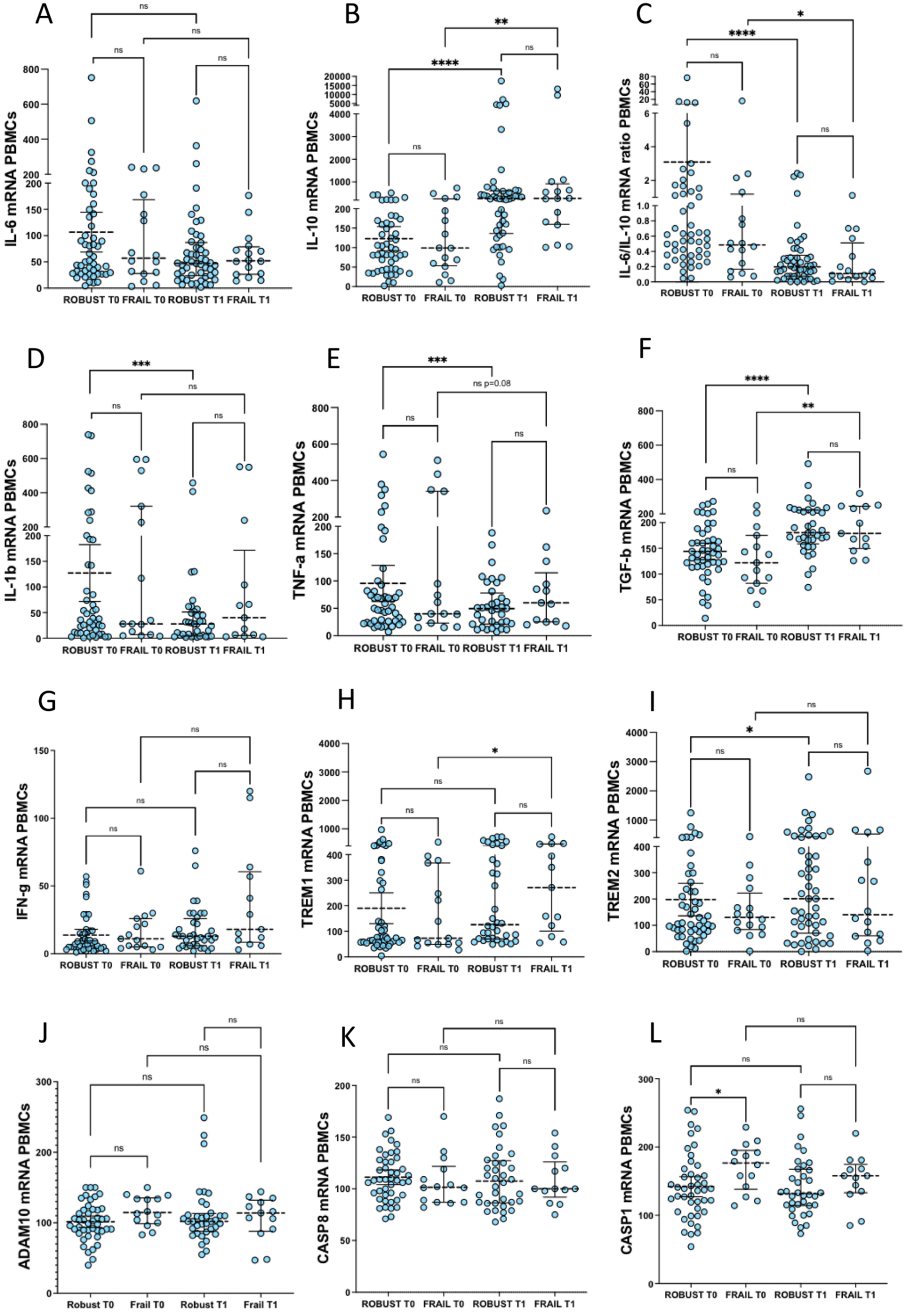
Effects of the VIVIFRAIL exercise protocol on relative PBMCs' mRNA expression of inflammatory mediators. Data were analyzed using the Wilcoxon matched‐pairs signed rank (within‐group repeated measures, T0 vs. T1) and the Mann–Whitney test (Robust vs. Frail). Values are shown as median and interquartile range (IQR: 25‐75th percentile). *p < 0.05, **p < 0.01, ***p < 0.001, ****p < 0.0001.

After physical activity, the PBMC's mRNA of *IL‐10* was potently increased (robust *p* < 0.0001, frail *p* < 0.01) (Figure 3B) whereas the *IL‐6/IL‐10* ratio was decreased in both robust and frail subjects (robust *p* < 0.0001, frail *p* < 0.05) (Figure 3C). *IL‐1b* and *TNF‐a* mRNAs were also decreased in robust subjects (*p* < 0.001) (Figure 3D,E), while a downward trend for *TNF‐a*, which approached statistical significance, was observed in the frail (Figure 3E). Contrariwise, the mRNA of *TGF‐b* was increased in both groups (robust *p* < 0.0001, and frail *p* < 0.01) (Figure 3F). Moreover, frail individuals featured an increase in *TREM1* mRNA (*p* < 0.05) (Figure 3H), while robust individuals showed an increase in *TREM2* mRNA (*p* < 0.05) (Figure 3I). *CASP1* expression was higher in frail compared with robust subjects (Figure 3L). As for its association with HG at T0, sex‐related differences were also observed. In fact, females showed lower *CASP1* mRNA compared to males, which approached statistical significance at T0 (*p* = 0.06 for robust, *p* = 0.08 for frail) and was consistent at T1 (*p* < 0.01 for robust) (Figure S2B). However, physical activity did not produce significant effects on *CASP1* expression (Figure 3L), even when stratifying according to sex (Figure S3B).

**FIGURE 4:**
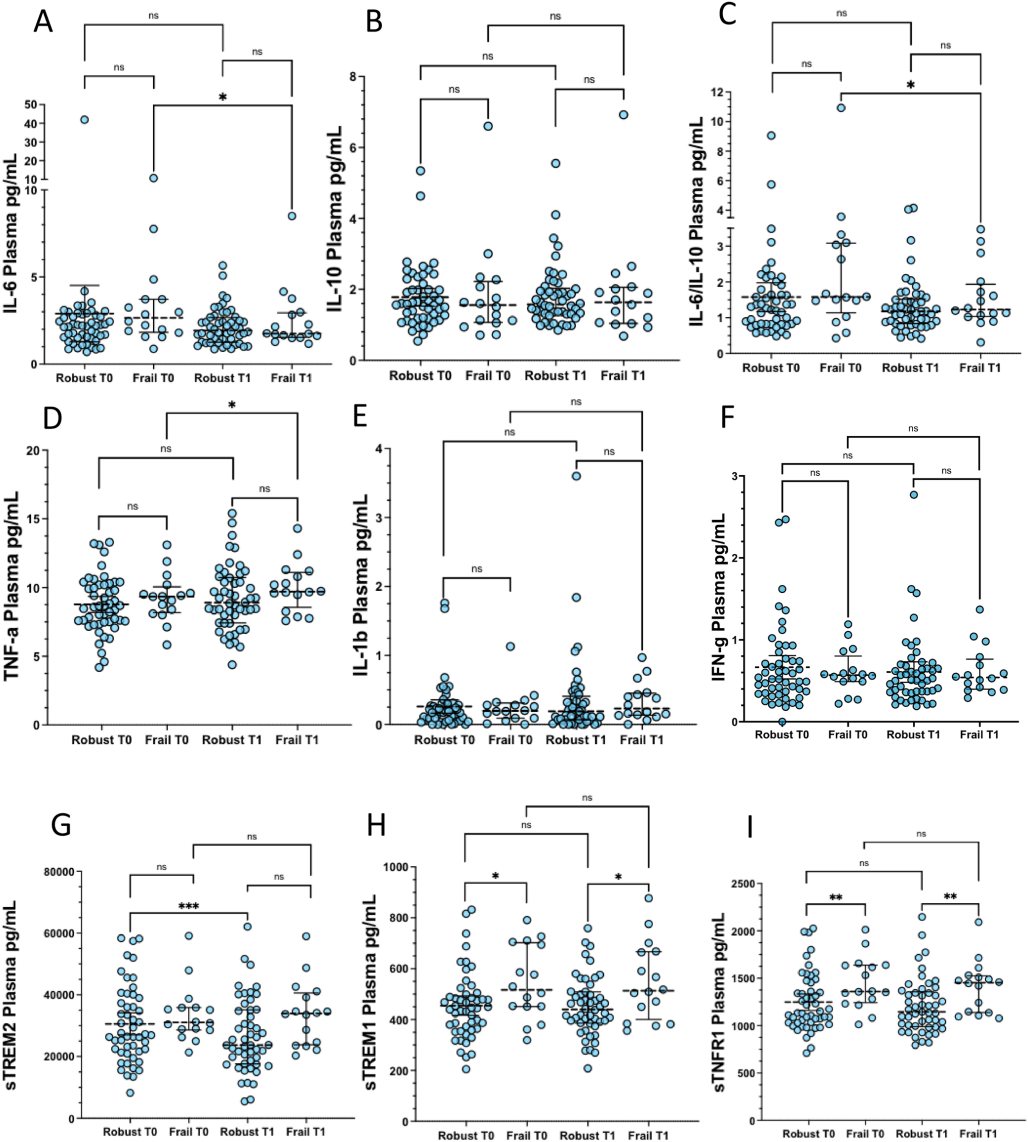
Effects of VIVIFRAIL exercise protocol on the plasmatic concentrations of inflammatory mediators. Data were analyzed using the Wilcoxon matched‐pairs signed rank (within‐group repeated measures, T0 vs. T1) and the Mann Whitney test (Robust vs. Frail). Values are shown as median and interquartile range (IQR: 25‐75th percentile). *p < 0.05, **p < 0.01, ***p < 0.001.

After physical activity, the plasmatic concentration of IL‐6 and the IL‐6/IL‐10 ratio was reduced in frail subjects specifically (*p* < 0.05) (Figure 4A,C), which was paralleled by a slight increase in TNF‐a (*p* < 0.05) (Figure 4D). The increase in plasmatic IL‐6 differed significantly in frail compared with robust subjects (Figure S4). Instead, the plasmatic concentration of sTREM2 was reduced in robust individuals specifically (*p* < 0.001) (Figure 4G). Plasmatic concentrations of sTREM1 and sTNFR1 were higher in frail vs. robust subjects at both T0 and T1 (*p* < 0.05 sTREM1, and *p* < 0.01 sTNFR1), while they were not significantly altered by physical activity in either group (Figure 4H,I).

### 3.5 Effects of VIVIFRAIL Exercise Protocol on Circulatory Mediators of the Muscle–Brain Axis

**FIGURE 5:**
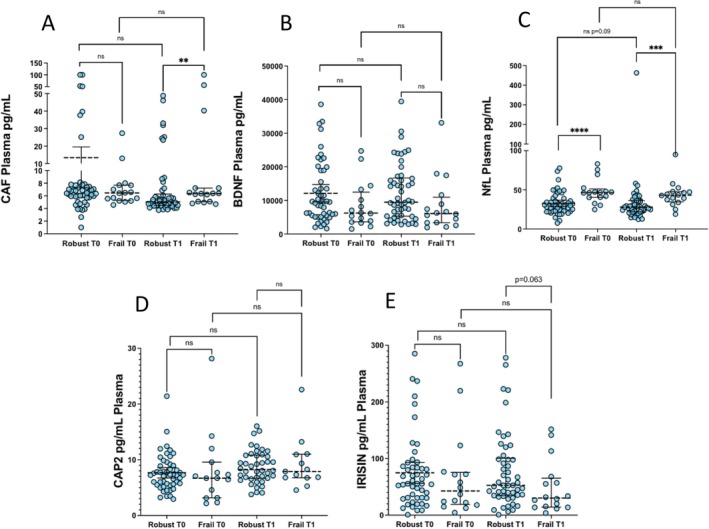
Effects of the VIVIFRAIL exercise protocol on plasmatic/serum concentrations of muscle‐brain axis' mediators. Data were analyzed using the Wilcoxon matched‐pairs signed rank (within‐group repeated measures, T0 vs. T1) and the Mann–Whitney test (Robust vs. Frail). Values are shown as median and interquartile range (IQR: 25‐75th percentile). **p < 0.01, ***p < 0.001, ****p < 0.0001.

After physical activity, no alterations in the plasmatic concentrations of CAF, BDNF, NfL, CAP2, or Irisin were observed (Figure 5A–E). A downward trend that approached statistical significance was observed for NfL in robust subjects (Figure 5C). NfL was higher in frail compared to robust subjects at both T0 (*p* < 0.0001) and T1 (*p* < 0.001). At T1, plasmatic CAF levels were higher in frail compared to robust individuals (*p* < 0.01) (Figure 5A), and an opposite trend that approached statistical significance (*p* = 0.063) was observed for Irisin (Figure 5E). Irisin, which was significantly associated with HG in the robust group at T0, was indeed higher in robust males compared to robust females at both T0 (*p* < 0.05) and T1 (*p* < 0.01) (Figure S5).

### 3.6 Correlations Among VIVIFRAIL Protocol‐Induced Variations in Clinical Scores and Biological Mediators

**FIGURE 6:**
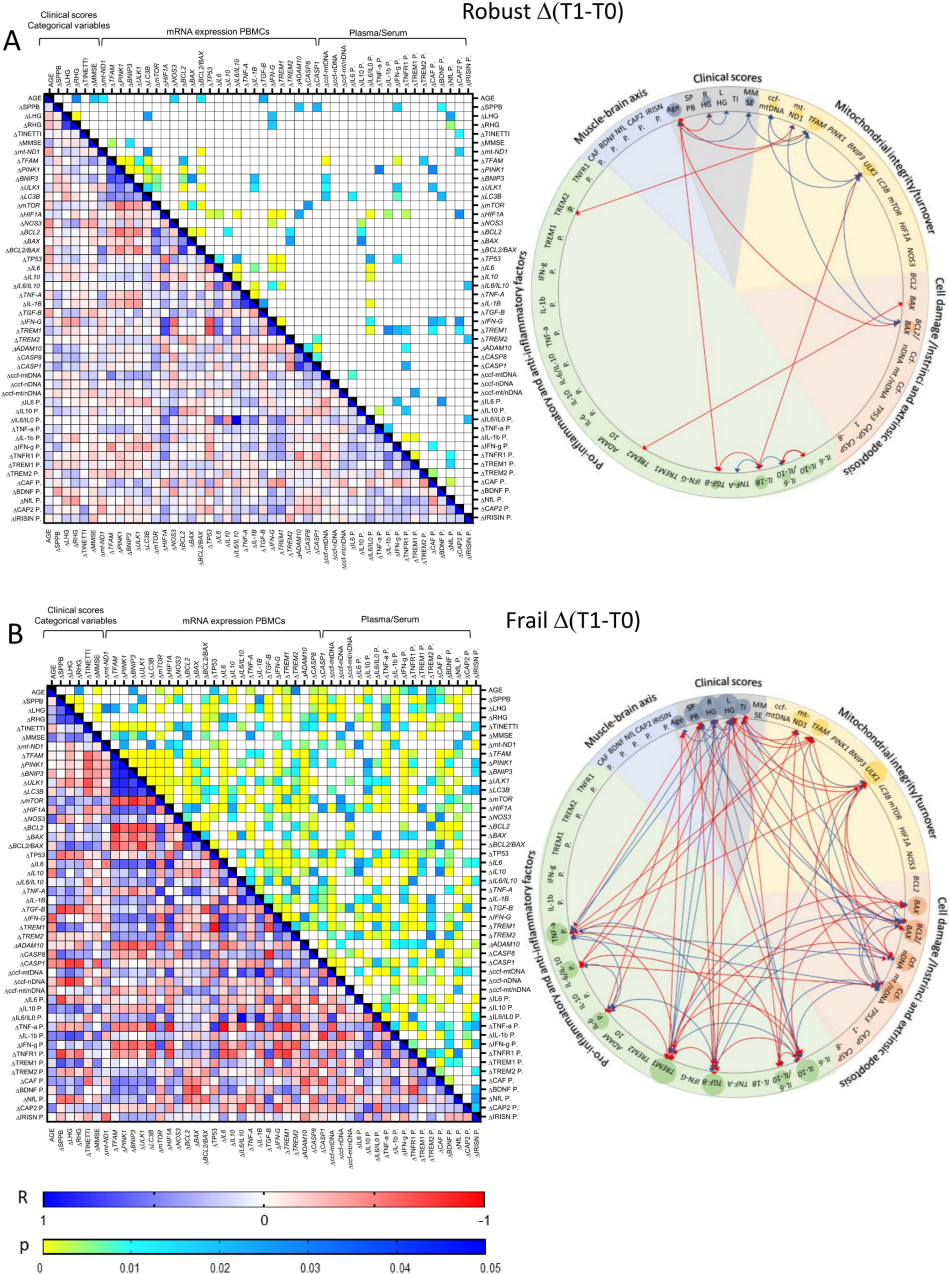
Spearman's correlations among VIVIFRAIL protocol‐induced variations (Δ, T1‐T0) in clinical scores, mitochondrial, apoptotic, inflammatory, and neuro/muscular mediators in robust (A) and frail (B) individuals. Heatmaps show Spearman's R correlations and related *p* values. The cartoons provide a summary of inter‐ and intra‐pathway positive (blue) and negative (red) associations. LHG, Left Handgrip; RHG, Right Handgrip; TI, Tinetti.

In robust individuals (Figure 6A), no associations emerged between the increases in physical scores and any of the biological mediators that were significantly modified following the exercise protocol. Some associations emerged between SPBB increase and variations in PBMCs mRNA of *ADAM10* (*R* = 0.38, *p* < 0.05), *CASP1* (*R* = 0.37, *p* = < 0.05), plasmatic IFN‐g (*R* = 0.32, *p* < 0.05), and BDNF (*R* = 0.36, *p* = 0.01). Likewise, significant associations emerged between the increase in HG scores and variations in PBMCs' mRNA of *LC3B* (*R* = −0.30, *p* < 0.05) and *IFN‐g* (*R* = −0.38, *p* < 0.05), as well as plasmatic IL‐1b (*R* = −0.30, *p* < 0.05) and NfL (*R* = −0.39 and −0.29, *p* < 0.05 and *p* < 0.01). However, none of these markers was significantly altered by the physical intervention (Figures 2, 3, 4). Interestingly, the increases in HG scores, as well as *mt‐ND1*, *BCL‐2/BAX*, and *TGF‐b* were negatively associated with age (HG, *R* = −0.29, *p* < 0.05; *mt‐ND1*, *BCL‐2/BAX*, *TGF‐b*, *R* = −0.35, *p* < 0.05). As for associations among biological mediators that varied significantly after the exercise protocol, a simplified network of interactions emerged: variations in components of the autophagy/mitochondrial pathway (*ULK1*, *TFAM*, *mt‐ND1*, and ccf‐mtDNA), along with the plasmatic cytokine IL‐1b were the most represented, followed by variations in the apoptotic index *Bcl2/BAX* ratio and inflammatory markers (*IL‐6/IL‐10* ratio, *TGF‐b*, *IL‐10*, *TNF‐a*, *BAX*, *TREM2*, and plasmatic sTREM2) (Figure 6A). In particular, the reduction in *ULK1* mRNA was associated with (i) the reduction in plasmatic ccf‐mtDNA (*R* = 0.36, *p* < 0.01) and *TFAM* mRNA (*R* = 0.36, *p* < 0.01), and (ii) the increase in *Bcl‐2/BAX* (*R* = −0.51, *p* < 0.001) and *IL‐1b* mRNA expression (*R* = −0.40, *p* < 0.01). Variations in *IL‐1b* expression were in turn associated with the increase in *TGF‐b* (*R* = −0.34, *p* < 0.05) and with a decrease in *IL‐6/IL‐10* mRNA ratio (*R* = 0.53, *p* < 0.001). The reduction in *TFAM* was associated with the reduction in ccf‐mtDNA (*R* = 0.51, *p* < 0.001), but also with an increase in plasmatic sTREM2 (*R* = −0.32, *p* < 0.05). Finally, the increase in *Bcl‐2/BAX* was associated with increased *mt‐ND1* (*R* = 0.52, *p* < 0.001), while *BAX* reduction was associated with increased *TREM2* mRNA expression (*R* = −0.30, *p* < 0.05) (Figure 6A).

In the frail group (Figure 6B), the increases in SPPB, Tinetti, and HG scores were associated with variations in several biological mediators that were indeed significantly modified after the physical intervention. In detail, SPPB increase was associated with variations in (i) HG scores (*R* = 0.7 and 0.6, *p* < 0.0001), (ii) PBMC's mRNA of autophagy/mitochondrial pathway mediators *ULK1* (*R* = −0.29, *p* < 0.05) and *Bcl‐2/BAX ratio* (*R* = 0.37, *p* < 0.01), (iii) PBMC's mRNA of inflammatory factors *IL‐10* (*R* = 0.49, *p* < 0.001), *TGF‐b* (*R* = 0.73, *p* < 0.0001), and *TREM1* (*R* = 0.65, *p* < 0.0001), (iv) plasmatic cell danger mediator ccf‐nDNA (*R* = −0.56, *p* < 0.0001) and inflammatory cytokines IL‐6 (*R* = −0.72, *p* < 0.0001) and TNF‐a (*R* = −0.39, *p* < 0.01). Contrarily to robust subjects, the increases in HG scores were associated positively with age (*R* = 0.30, *p* < 0.05). The increases in HG scores were also associated with variations in (i) PBMCs' mRNA of mitochondrial pathway mediators *mt‐ND1* (*R* = −0.31, *p* < 0.05), *TFAM* (*R* = −0.32, *p* < 0.05), *ULK1* (*R* = −0.47, *p* < 0.001), *Bcl‐2/BAX ratio* (*R* = 0.44 and 0.66, *p* < 0.01 and *p* < 0.0001), (ii) PBMC's mRNA of inflammatory factors *TGF‐b* (*R* = −0.62, *p* < 0.0001), *TREM1* (*R* = 0.49 and 0.43, *p* < 0.01 and *p* < 0.001), (iii) ccf‐nDNA (*R* = −0.61 and − 0.80, *p* < 0.0001), and inflammatory cytokines IL‐6 (*R* = −0.39 and −0.20, *p* < 0.001) and TNF‐a (*R* = −0.32, *p* < 0.05). The increase in Tinetti score was associated (i) negatively with PBMC's mRNA of autophagy/mitochondrial pathway mediators *TFAM* (*R* = −0.60, *p* < 0.0001), and *ULK1* (*R* = −0.70, *p* < 0.0001), and positively with *Bcl‐2/BAX ratio* (*R* = 0.56, *p* < 0.0001), (ii) negatively with PBMCs' mRNA of inflammatory index *IL‐6/IL‐10* (*R* = −0.40, *p* < 0.01) and plasmatic inflammatory factors IL‐6/IL‐10 (*R* = −0.40, *p* < 0.01) and TNF‐a (*R* = 0.30, *p* < 0.05). Finally, the increases in *IL‐10* and *TREM1* mRNA were negatively associated with age (*R* = −0.51, *p* < 0.001, *R* = −0.41, *p* < 0.01, respectively). Associations among variations in biological mediators produced a complex network of interactions featuring a major and quite equal contribution of the autophagy/mitochondrial pathway (*ULK1*, *TFAM*, *mt‐ND1*), cell danger/death (ccf‐nDNA, and ccf‐mt/nDNA ratio, *BAX*, *Bcl2/BAX*), and inflammatory mediators (*IL‐10, IL‐6/IL‐10* ratio, *TGF‐b*, *TREM1*, IL‐6, IL‐6/IL‐10, and TNF‐a) (Figure 6B).

Finally, in the frail group, variations in the plasmatic IL‐6 were negatively associated with variations of PBMCs' *IL‐6* mRNA, while a positive correlation emerged between variations in TREM1 mRNA expression and its plasmatic concentration (Figure 6B and File S1). Instead, the mitochondrial intra‐pathway associations observed at T0 were recapitulated also for their variations, with positive associations between *TFAM*, and *ULK1*, and their negative associations with *Bcl‐2/BAX* in both robust and frail individuals (Figure 6A,B and File S1).

## 4 Discussion

Physical exercise is now increasingly accepted as a cornerstone of intervention in frail people, as it might contribute to mitigating the adverse effects of aging. In agreement with previous findings (Agostini et al. 2023; Petrella et al. 2021), here we confirm that the home‐based, VIVIFRAIL multicomponent exercise protocol positively affects the functional status in robust and especially frail old individuals. The biological effects of the physical intervention were associated with a remodeling of mitochondrial, inflammatory, and cell danger/apoptotic pathways in the peripheral immune system, both intracellularly in PBMCs and at the circulatory level (plasma/serum). However, such effects varied in part according to the baseline (T0) physical status and age, which were associated with different metabolic and inflammatory profiles observed in robust vs. frail groups, providing some interesting insights into the biological mechanisms underpinning the different trajectories of aging.

In fact, the associations in robust individuals at T0 (Figure 1A) indicate that, albeit being functionally classified as robust, age in such subjects is associated with lower physical scores (Tinetti) and lower neurotrophic support (plasmatic BNDF). Moreover, physical scores (SPPB, HG, and/or Tinetti) were associated with a transcriptional and circulatory systemic profile characterized by (i) active anti‐inflammatory response (*IL‐10*), (ii) active release of pleiotropic mediators of the muscle‐brain axis (Irisin), and (iii) limited mitochondrial biogenesis and apoptotic response (*mt‐ND1* and *Bcl‐2/BAX*), suggesting a dynamic coordination that probably is in place to grant systemic homeostasis and physical functions (Figure 1A).

Associations in frail individuals at T0 (Figure 1B) suggest that Tinetti score is negatively associated with age, as in robust ones. However, in the frail, physical scores (SPPB, HG, and/or Tinetti) were instead associated with (i) abnormal mitochondrial dynamics consisting of unbalanced mitochondrial biogenesis and mitophagy (*TFAM, BNIP3*, and *mTOR*), and (ii) impaired ability to properly cope with inflammation (IL‐6/IL‐10 ratio, IL‐1b, *CASP‐1*). Compared to robust individuals, inter‐ and intra‐pathway associations in frail subjects depicted a network of less numerous interactions (Figure 1B). Mediators of the muscle‐brain axis (mostly CAP2, BDNF, and CAF) were moderately represented in the frail group, correlating with cell death, and inflammatory pathway mediators (Figure 1B). Interestingly, CAP2 correlated positively with the expression of anti‐inflammatory cytokines (*IL‐10*, and *TGF‐b*), and negatively with pro‐inflammatory and pro‐apoptotic factors (*IL‐6/IL‐10* mRNA ratio, *CASP8*, and *BAX*). CAP2 is known for its key role in regulating actin dynamics, cell signaling, and immune function. Although its role in plasma is still unclear, recent studies documented an inverse association between circulating CAP2 concentration and frailty (Pelucchi et al. 2023). At baseline, frail subjects also showed higher levels of *CASP1*, sTREM1, sTNFR1, and NfL compared to robust ones (Figures 3, 4, 5). Although age clearly influences baseline performance in both robust and frail individuals, as indicated by the associations between Tinetti scores and age at T0, these data suggest that physical frailty is a major contributor to the “inflammaging” phenotype characterizing frail subjects.

This was substantiated in part by the data on ccf‐mtDNA, which represents one of the most novel and important results of our study (Figure 2). In our cohort, ccf‐mtDNA was higher in robust compared to frail individuals at baseline, and it was consistently reduced in robust but not frail subjects after the VIVIFRAIL protocol (Figures 2A and S2). Ccf‐mtDNA levels in the serum of older adults were shown to be positively correlated with markers of “inflammaging” such as CRP, TNF‐a, sTNFR1, and IL‐6; however, conflicting results still exist about a possible association between ccf‐mtDNA levels and physical/cognitive status (Nidadavolu et al. 2023; Pinti et al. 2014). Beyond disease states, high levels of ccf‐mtDNA have been observed in various contexts, as healthy aging, representing a positive, adaptive response to inflammaging through maintenance of immune surveillance (Byappanahalli et al. 2023; Nidadavolu et al. 2023; Pinti et al. 2014). Accordingly, dysregulations of the mitochondria‐inflammation axis should not necessarily be considered a mere trigger of age‐related diseases but rather as an adaptive process capable of stimulating an appropriate anti‐inflammatory and metabolic response. In fact, the balances between inflammaging and “anti‐inflammaging,” along with the generation of new mitochondria (biogenesis), and the removal of damaged ones (mitophagy), are key factors that determine the trajectory of aging (Guilbaud, Sarosiek, and Galluzzi 2024).

Under these premises, high ccf‐mtDNA levels could indicate both active cellular and metabolic turnover, as well as mitochondrial quality control mechanisms, such as mitophagy. In fact, at T0, the levels of plasmatic ccf‐mtDNA in robust individuals were associated with both mitochondrial quality control/mitophagy markers (mRNA expression of *TFAM*, *ULK1*, *LC3B*) and inflammatory factors (mRNA expression of *TNF‐a*, and *IL‐1b*, and plasmatic IL‐1b) (Figure 1A), suggesting an active mitochondrial turnover profile that copes with immune functions during the normal aging process.

Intriguingly, such associations were not observed in frail subjects. In this frame, a few studies suggest that a reduction in ccf‐mtDNA levels may occur during aging and neurodegenerative diseases, which might be related to progressive cell dysfunction (Gambardella et al. 2019; Lazo et al. 2021).

Exercise has been shown to influence ccf‐mtDNA levels (Trumpff et al. 2021). While acute exercise can induce a temporary increase in ccf‐mtDNA and ccf‐nDNA (Atamaniuk et al. 2004; Trumpff et al. 2021) due to muscle damage and repair processes, regular physical activity can lead to a beneficial effect on mitochondrial turnover, potentially reflected in stable levels of both components that can be observed in active adults (Nasi et al. 2016), as observed in our robust subjects. Thus, the lower levels of ccf‐mtDNA detected in frail individuals at T0, coupled with the lack of ccf‐mtDNA changes after physical activity, might indicate reduced cellular metabolism and function in frail compared to robust individuals.

Another interesting finding of our study is that following physical activity, ccf‐nDNA levels were almost abolished in both robust and frail subjects (Figure 1B). Ccf‐nDNA may arise from cell death, tissue damage, or inflammation, and by acting as a DAMP, it sustains stress‐related and inflammatory responses contributing to frailty (Kananen et al. 2023).

Because of the dramatic reduction in ccf‐nDNA, the ccf‐mt/nDNA ratio (an index of cell integrity/metabolism mirrored by the mtDNA copies released from a dead/damaged cell) was consistently increased in both robust and frail individuals after physical activity (Figure 1C). However, caution is mandatory when interpreting such a result, as ccf‐nDNA copies were almost negligible at T1 compared to ccf‐mtDNA, and their ratio inevitably tends to abnormal values.

In both robust and frail individuals, increased PBMCs mRNA expression of the mitochondrially encoded gene *mt‐ND1* was detected following physical activity (Figure 2). This indicates increased mtDNA replicative capacity/integrity and mitochondrial biogenesis to support immune cell function.

At the same time, both robust and frail individuals showed reduced mRNA expression of *TFAM* and *ULK1*, two key regulators of mtDNA replication/transcription and autophagy/mitophagy (Figure 2). This seemingly contradictory *mt‐ND1* upregulation going along with *TFAM/ULK1* downregulation reflects complex regulatory mechanisms that are in place to ensure optimal and balanced mitochondrial function and that can be influenced by various factors such as cellular stress, energy demands, and adaptive/compensatory responses triggered by physical exercise.

The reduction of *BAX* mRNA, along with the increase in the *Bcl‐2/BAX* ratio detected after physical activity in both robust and frail subjects (Figure 2), indicates an adaptive response to maintain a functional immune cell population. These results suggest that despite the differences in functional state, robust and frail individuals show some similar changes in mitochondrial and cell survival systems following the exercise protocol.

This was partly reproduced for inflammatory pathways (Figures 3 and 4). After physical exercise, both robust and frail individuals showed increased mRNA expression of the anti‐inflammatory cytokines *TGF‐β* and *IL‐10*, along with reduced pro‐inflammatory *IL‐6/IL‐10* mRNA ratio (Figure 3). However, at the circulatory level, this was recapitulated in frail subjects only, who showed a slight, though significant reduction in plasmatic IL‐6 and IL‐6/IL‐10 ratio concentration (Figure 4). As no changes in IL‐6 gene expression in PBMCs were found after physical intervention, our data suggest that post‐transcriptional mechanisms occur to fine‐tune the circulating levels of this cytokine. On the other hand, the remarkable increase in *IL‐10* expression seen in PBMCs was not reflected in IL‐10 plasmatic concentrations in either group, suggesting an ongoing transcriptional anti‐inflammatory response that can be readily detected within immune cells but is temporarily uncoupled from cytokine production and release. Intriguingly, frail subjects also showed a slight upregulation of plasmatic TNF‐α, suggesting that the upregulation of the anti‐inflammatory cytokines *TGF‐β* and *IL‐10* might occur as a counter‐regulatory mechanism to balance pro‐inflammatory cytokines that are frequently detected after physical exercise (Pedersen and Hoffman‐Goetz 2000).

Another novel finding of our study is that robust individuals showed an increase in mRNA expression of *TREM2*, along with a concomitant decrease in its plasmatic soluble form sTREM2. An increase in sTREM2 was observed in the cerebrospinal fluid of patients with Alzheimer disease after a 16‐week physical intervention (Jensen et al. 2019). However, no studies to date have investigated the role of sTREM2 in healthy older individuals following physical activity. Given the anti‐inflammatory nature of TREM2 compared with its counterpart TREM1, it may be tempting to speculate that the shedding of the TREM2 receptor is probably hindered by physical intervention to promote its intracellular storage.

Finally, albeit a downward trend for plasmatic NfL, which approached statistical significance, in the robust physical exercise did not produce significant changes in the plasmatic levels of any muscle‐brain mediators in either group (Figure 4).

In robust individuals, none of the biological mediators that varied significantly following the exercise protocol were associated with the increase in physical scores (Figure 6A). However, changes in *mt‐ND1, BCL‐2/BAX, and TGF‐b* were negatively associated with age, suggesting that chronological age primarily influences exercise‐induced biological modulations in the robust group.

Associations among mitochondrial modulators suggest that the reduction/normalization of autophagy/mitophagy‐related genes that takes place following physical activity is an attempt to balance mitochondrial removal and biogenesis while maintaining mitochondrial function and stability. This potentially leads to reduced extrusion of ccf‐mtDNA that occurs alongside reduced apoptosis. These modulations were associated with both pro‐inflammatory (increase of IL‐1β) and anti‐inflammatory mechanisms (increased *TGF‐b*, reduced *IL‐6/IL‐10* mRNA ratio). Intracellular *TREM2* expression was also increased in association with a downregulation in cell death pathways.

Remarkably, in frail subjects, the improvements in physical performance, muscle strength, as well as balance and gait were indeed linked to several biological modulations, namely the reduction of both cellular stress (reduced markers of autophagy/mitophagy *ULK1*, and *TFAM* mRNAs) and inflammation (reduced plasmatic IL‐6, and IL‐6/IL‐10 ratio both at mRNA and plasmatic levels), along with a potentiation of cell survival and anti‐inflammatory mechanisms (increased *BCL‐2/BAX* and *TGF‐b* mRNAs) (Figure 6B). Interestingly, negative associations between variations in physical scores (SPPB, HG, and/or Tinetti) and pro‐inflammatory factors like plasmatic TNF‐a and *TREM1* mRNA were also observed, suggesting the occurrence of adaptive inflammatory mechanisms following the exercise protocol. Moreover, in frail subjects, contrarily to robust ones, the increase in HG scores was negatively associated with age. Again, changes in a few biological mediators (*IL10* and *TREM1* mRNAs) were associated with age besides physical scores (HG and SPPB).

These data suggest that age plays a role in determining the response to intervention with regard to both functional parameters and biological mediators in both groups. However, the numerous significant correlations found in the frail group between improvements in functional parameters and changes in biological mediators seem to indicate that the state of physical frailty profoundly influences how functional changes are reflected at the biological level.

We wish to point out that our study has several limitations. Firstly, considering the single‐center nature of the study and the relatively small and heterogeneous sample size, primarily due to the difficulty in enrolling frail subjects, our results need to be confirmed in larger cohorts. Moreover, as expected, individuals with physical frailty are older than the robust ones. While age clearly plays a role in baseline functional capacity and some aspects of responsiveness to exercise, the more consistent links between biological modulations and functional improvements observed in the frail group indicate that frailty status is a key modulator of these changes. A combined influence of both factors should be considered, with frailty likely modifying the relationship between age, biological mediators, and functional outcomes. In addition, the study lacks follow‐up data because of the COVID‐19 pandemic, which prevented longer‐term evaluations after the intervention protocol. Finally, our study includes secretome analysis from plasma/serum and quantification of mRNA but not protein expression from PBMCs. This would have added value to our findings, as mRNA expression changes may often occur as a compensatory response to altered proteostasis. However, given the increasing clinical relevance of frailty and the importance of preventing its progression to sarcopenia, our exploratory study may serve as a basis for larger confirmatory investigations.

## 5 Conclusions

Besides confirming the efficacy of the VIVIFRAIL multicomponent exercise protocol on functional outcomes in the older population, the present study highlights previously undescribed modulations and interactions within mitochondrial, cell death, and inflammatory pathways occurring at intracellular and circulatory levels. Key findings from easily accessible biological samples, including plasma, serum, and PBMCs, revealed enhanced mitochondrial biogenesis (increased *mt‐ND1*), optimized autophagy/mitophagy (reduction of *TFAM*, and *ULK1*), improved cell survival (increased *BLC‐2/BAX*, reduced ccf‐nDNA), and decreased expression of pro‐inflammatory mediators (decreased *IL6/IL10* ratio, increased *IL‐10*, and *TGF‐b*). Distinct adaptations were also observed, namely, a reduction of plasmatic ccf‐mtDNA and sTREM2, along with upregulation of *TREM2* in robust subjects, and a reduction of IL6 and IL6/IL10 ratio, along with upregulation of TNF‐a and *TREM1* in frail subjects. This suggests that beyond straightforward reduction of inflammation, physical exercise triggers divergent and complex adaptation mechanisms related to frailty status. Although confirmatory studies on larger cohorts are necessary, our study is expected to contribute to a better understanding of both the biological bases of healthy and unhealthy aging, as well as the cellular and molecular mechanisms underpinning the benefits of physical activity in the aging population.

## Author Contributions

Conceptualization, F.L., E.F., M.B., B.A.; methodology, F.L., E.F., P.O., G.A.M., C.F., L.D'A., E.M.; software, F.L., E.F.; validation, F.R.G., M.C., E.M., M.B., B.A.; formal analysis, F.L., E.F.; investigation, F.L., E.F., P.O., G.A.M., L.D'A.; resources, T.L., M.C., M.B., B.A.; data curation, F.L., E.F., M.B., B.A.; writing – original draft preparation, F.L., E.F.; writing – review and editing, F.L., E.F., L.D'A., E.M., M.B., B.A.; supervision, M.B., B.A.; funding acquisition, M.C., M.B., B.A. All authors have read and agreed to the present version of the manuscript.

## Ethics Statement

The study was performed in accordance with the Declaration of Helsinki. An informed consent was signed by all the participants according to a protocol approved by the ethics committee of Fondazione IRCCS Ca’ Granda, Ospedale Maggiore Policlinico (n#343_2018).

## Conflicts of Interest

The authors declare no conflicts of interest.

## Supporting information


Appendix S1.

Appendix S2.

## Data Availability

The data that supports the findings of this study are available in the [Link], [Link] of this article.
