# Supplementary material for: Effects of the VIVIFRAIL Exercise Protocol on Circulatory and Intracellular Peripheral Mediators Bridging Mitochondrial Dynamics and Inflammation in Robust and Frail Older People
Source: Aging Cell. 2025 Mar 4;24(6):e70029. doi: 10.1111/acel.70029 (PMC12151904; doi:10.1111/acel.70029)
Supplement: Supplementary file 2 — Appendix S2. [file ACEL-24-e70029-s001.pdf]

SUPPLEMENTARY MATERIAL. Effects of the VIVIFRAIL© exercise protocol on circulatory and intracellular peripheral mediators bridging mitochondrial dynamics and inflammation in robust and frail older people

Biological Pathway	Plasma/Serum	PBMC's mRNA
**Muscle-brain axis**	• Irisin<br>• C-terminal Agrin Fragment (CAF)<br>• Brain-Derived Nerve Factor (BDNF)<br>• Neurofilament Light chain (NfL)<br>• Cyclase-associated protein 2 (CAP2)	
**Inflammation**	• Interleukin (IL)-6<br>• IL-10<br>• IL-1b<br>• Tumor Necrosis Factor-α (TNF- α)<br>• Interferon gamma (IFN-g)<br>• TNF- α Receptor 1 (TNFR1)<br>• Soluble Triggering receptor expressed on myeloid cells 1 (sTREM1) and 2 (sTREM2)	• *IL-6*<br>• *IL-10*<br>• *IL-1b*<br>• *TNF- α*<br>• *IFN-g*<br>• *Transforming growth factor beta 1 (TGF-β1), hereafter referred to as TGF-β*<br>• *TREM1*<br>• *TREM2*<br>• *A Disintegrin and metalloproteinase-domain containing protein 10 (ADAM10)*<br>• *Caspase 1 (CASP1) and 8 (CASP8)*
**Mitochondria/ mitophagy-**<br><br>**cell danger/apoptosis-**<br><br>**hypoxia**	• Circulating cell-free mtDNA (ccf-mtDNA)<br>• Circulating cell-free nuclear DNA (ccf-nDNA)	• *Transcription factor A, mitochondrial (TFAM)*<br>• *Mitochondrially encoded NADH dehydrogenase 1 (mt-ND1)*<br>• *Phosphatase and tensin homologue-induced kinase 1 (PINK1)*<br>• *BCL2 -interacting protein 3 (BNIP3)*<br>• *Unc-51 like autophagy activating kinase 1 (ULK1)*<br>• *Mechanistic/mammalian target of rapamycin (mTOR)*<br>• *Microtubule-associated protein 1B-light chain 3 (LC3B)*<br>• *B-cell leukemia/lymphoma 2 protein (Bcl-2)*<br>• *Bcl-2-associated x protein (BAX)*<br>• *Tumor protein p53 (TP53)*<br>• *Hypoxia inducible factor 1 subunit alpha (HIF1A)*<br>• *Nitric oxide synthase 3 (NOS3)*

Supplementary Table 1. List of biological mediators analysed in Plasma/Serum and/or PBMCs at T0 and T1.

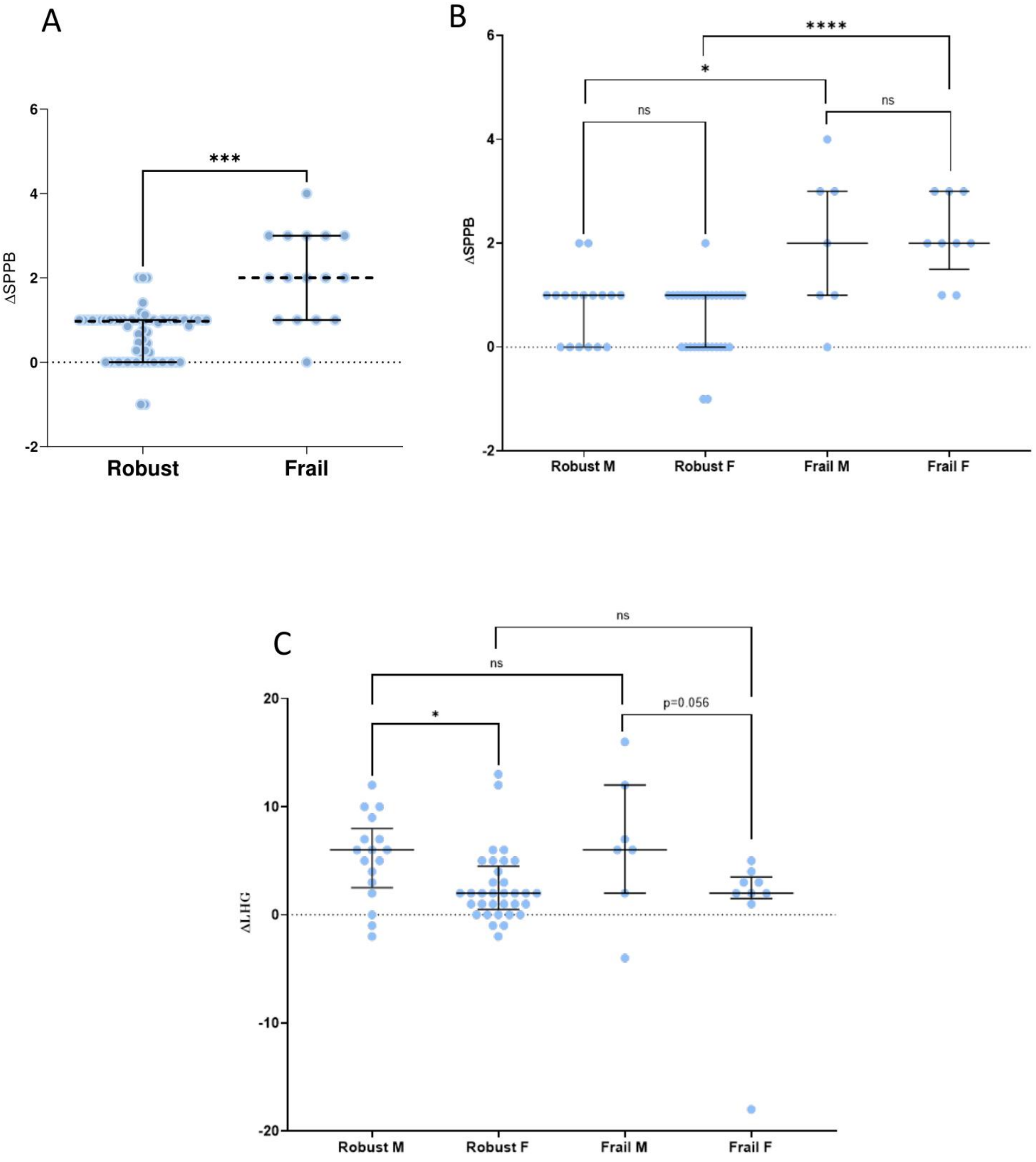

Supplementary Figure S1. Graphs show statistically significant T1-T0 variations (delta, Δ) of **A**) SPPB in Robust, and Frail, **B**) SPPB in Robust, and Frail Males (M), and Females (F), **C**) Left HG (LGH) in Robust, and Frail Males (M), and Females (F). Data were analysed using the Mann Whitney test. Values are shown as Median and Interquartile Range (IQR: 25-75th percentile). *p<0.05; ***p<0.001; ****p<0.0001.

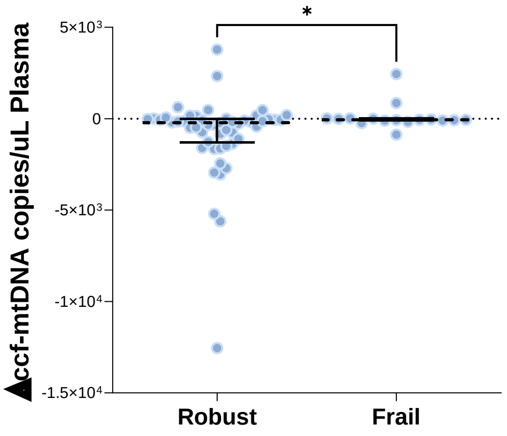

Supplementary Figure S2. The graph shows statistically significant T1-T0 variation (delta, Δ) of plasmatic ccf-mtDNA copies in Robust, and Frail. Data were analysed using the Mann Whitney test. Values are shown as Median and interquartile range (IQR: 25-75th percentile). *$p<0.05$.

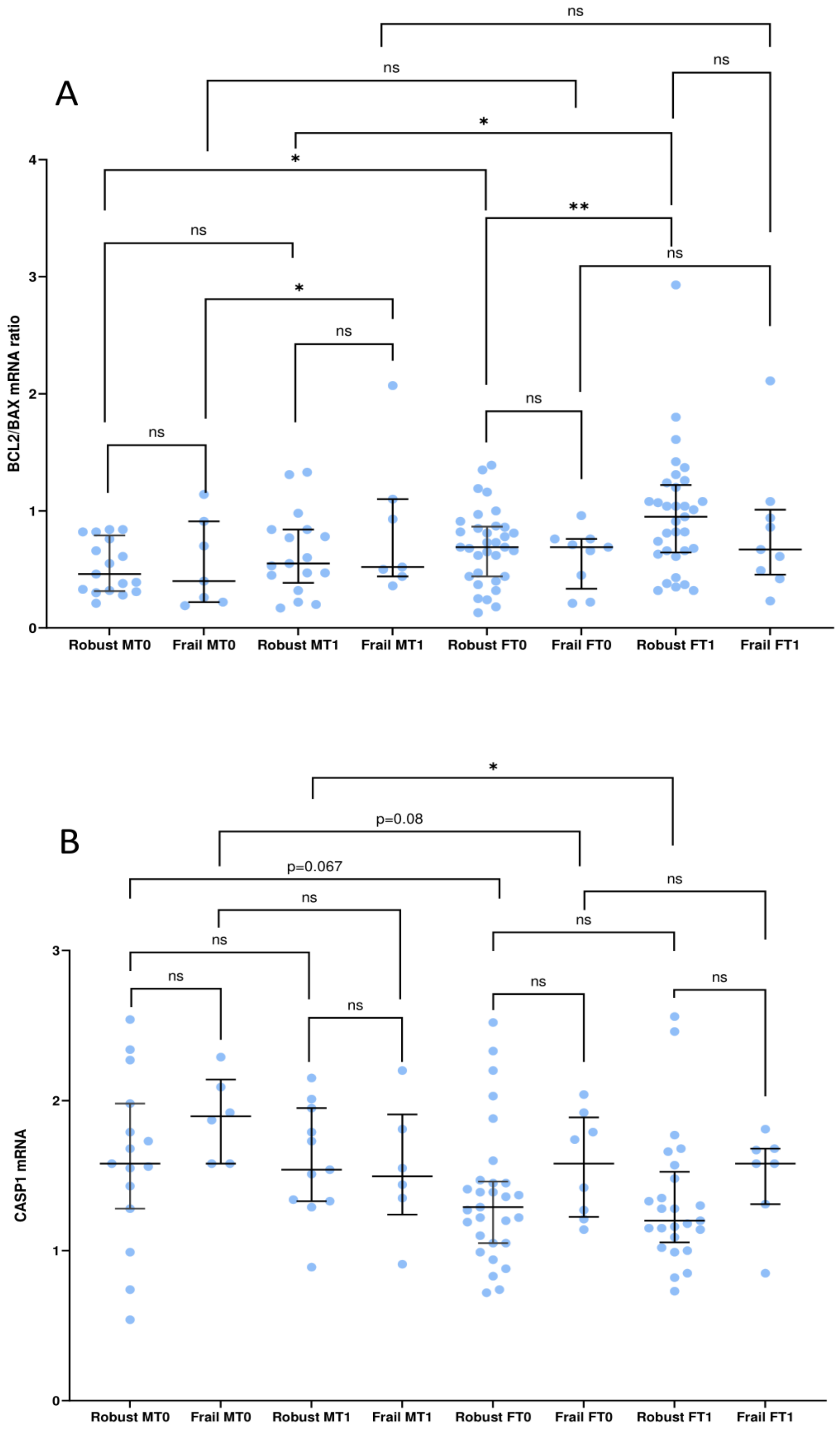

Supplementary Figure S3. Graphs show the relative, PBMC's mRNA expression of **A**) *BCL-2/BAX* ratio, and **B**) *CASP1* in Robust and Frail, Males (M), and Females (F), at T0 and T1. Data were analysed using the Wilcoxon matched-pairs signed rank (within-group repeated measures, T0 vs T1) and the Mann Whitney test (Robust vs Frail, M vs F). Values are shown as median and interquartile range (IQR: 25-75th percentile). *$p<0.05$, **$p<0.01$, ***$p<0.001$, ****$p<0.0001$.

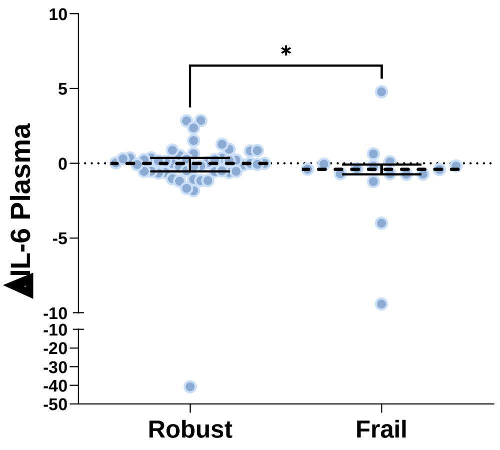

Supplementary Figure S4. The graph shows statistically significant T1-T0 variation (delta, Δ) of plasmatic IL-6 concentration in Robust, and Frail. Data were analysed using the Mann Whitney test. Values are shown as Median and interquartile range (IQR: 25-75th percentile). *p<0.05.

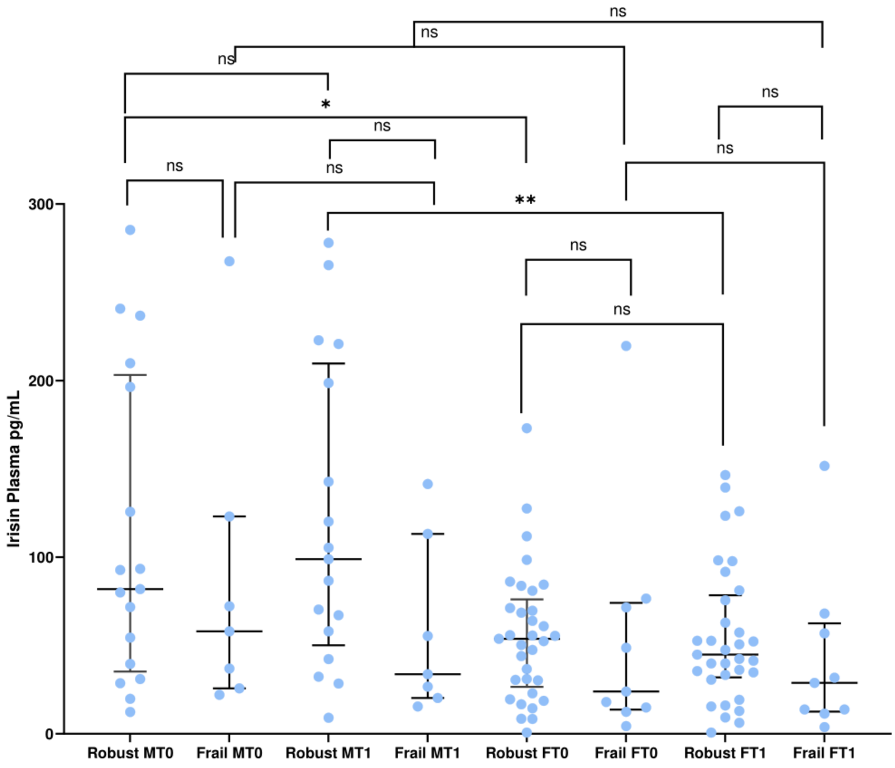

Supplementary Figure S5. The graph shows circulating Irisin concentration in Robust and Frail, Males (M), and Females (F), at T0 and T1. Data were analysed using the Wilcoxon matched-pairs signed rank (within-group repeated measures,

T0 vs T1) and the Mann Whitney test (Robust vs Frail, M vs F). Values are shown as median and interquartile range (IQR: 25-75th percentile). *$p<0.05$, **$p<0.01$.
